# Role of T Cells in Type 2 Diabetic Nephropathy

**DOI:** 10.1155/2011/514738

**Published:** 2011-10-19

**Authors:** Chia-Chao Wu, Huey-Kang Sytwu, Kuo-Cheng Lu, Yuh-Feng Lin

**Affiliations:** ^1^Division of Nephrology, Department of Medicine, Tri-Service General Hospital, National Defense Medical Center, Taipei 114, Taiwan; ^2^Graduate Institute of Microbiology and Immunology, National Defense Medical Center, Taipei 114, Taiwan; ^3^Department of Medicine, Cardinal Tien Hospital, School of Medicine, Fu Jen Catholic University, Chung-Cheng Road, Hsin-Tien, New Taipei City 231, Taiwan; ^4^Division of Nephrology, Department of Medicine, Shuang-Ho Hospital, Taipei Medical University, Taipei 110, Taiwan

## Abstract

Type 2 diabetic nephropathy (DN) is the most common cause of end-stage renal disease and is increasingly considered as an inflammatory disease characterized by leukocyte infiltration at every stage of renal involvement. Inflammation and activation of the immune system are closely involved in the pathogenesis of diabetes and its microvascular complications. Macrophage has been well recognized to play an important role in type 2 DN, leukocyte infiltration, and participated in process of DN, as was proposed recently. Th1, Th2, Th17, T reg, and cytotoxic T cells are involved in the development and progression of DN. The purpose of this review is to assemble current information concerning the role of T cells in the development and progression of type 2 DN. Specific emphasis is placed on the potential interaction and contribution of the T cells to renal damage. The therapeutic strategies involving T cells in the treatment of type 2 DN are also reviewed. Improving knowledge of the recognition of T cells as significant pathogenic mediators in DN reinforces the possibility of new potential therapeutic targets translated into future clinical treatments.

## 1. Introduction

Diabetes mellitus (DM) is a complex syndrome characterized by absolute or relative insulin deficiency leading to hyperglycemia and an altered metabolism of glucose, fat, and protein. These metabolic dysfunctions are pathologically associated with specific microvascular diseases and various characteristic long-term complications, including diabetic neuropathy, nephropathy, and retinopathy. Diabetic nephropathy (DN), affecting more than one third of patients with type 1 DM and up to 25% of all patients with type 2 DM, is an extremely common complication of DM that profoundly contributes to patient morbidity and mortality [1–4]. Diabetic nephropathy is a leading cause of chronic kidney disease, resulting in end-stage renal disease (ESRD) which has became a major problem facing human health worldwide [1–4]. Rapidly increasing rates of DM with profound consequences of DN are the primary reason for this worldwide increase. Diabetic nephropathy (DN) is characterized as pathological findings of hypertrophy of glomerular structures, thickening of the basement membrane, and accumulation of extracellular matrix (ECM) components. Multiple mechanisms contribute to the development and outcomes of DN, such as an interaction between metabolic abnormalities, hemodynamic changes, genetic predisposition and inflammatory milieu, and oxidative stress, constituting a continuous perpetuation of injury factors for the initiation and progression of both of DM and DN [5]. Traditionally, metabolic and hemodynamic factors are the main causes of renal lesions in patients with type 2 DM and DN, both considered nonimmune diseases [6–8]. However, recent studies have shown that chronic inflammation is associated with the development and progression of type 2 DM, implying that immunologic and inflammatory mechanisms may play a pivotal role in the disease process [9–11]. Furthermore, increased infiltration of monocytes/macrophages and activated T lymphocytes, as well as augmented expression of inflammatory cytokines in the kidneys have also been found in patients with DN [9–11]. Serial research has demonstrated that DN is a metabolic and hemodynamic disorder, with inflammation playing a vital role in the process [12, 13]. Type 1 DM is an autoimmune disease, and the role of T cell has been well recognized in the disease process. However, the role of T cells in type 2 DM is still debated. Several animal models are ideal for type 2 DM and DN that are extremely similar to that of humans and provide the tools necessary to investigate associated mechanisms [14–16]. The purpose of this review is to assemble current information concerning the role of T cells in the development and progression of type 2 DN, as evidenced from animal models related to human disease. Specific emphasis is placed on the potential interaction and contribution of the T cells to kidney damage. In addition, we also review the therapeutic strategies involving T cells in the treatment of type 2 DN.

## 2. T Cells, Metabolic Syndrome, and Type 2 DM

### 2.1. Adaptive T Cell Immune Response

The immune system is composed of innate and adaptive immunity. Innate immune system activation associated with chronic inflammation has been revealed to participate in the pathogenesis of type 2 diabetes and its complications [11]. It is widely recognized that adaptive immunity CD4^+^ T cells can be differentiated into T-helper 1 (Th1), Th2, Th17, and Treg according to their cytokine profiles [17, 18]. Th1 cells produce large quantities of interferon-*γ* (IFN-*γ*), induce delayed hypersensitivity reactions, activate macrophages, and promote cell-mediated immunity. Th2 cells produce mainly interleukin-4 (IL-4), induce IgE production, suppress Th1 cell activation, and contribute to humoral immunity [17, 18]. Th17, the recently discovered CD4^+^ effector T cell lineage distinct from Th1 and Th2, is a third distinct subset of T helper cells preferentially producing interleukin-17, but not IFN-*γ* or IL-4. We now have three types of effector helper T cells: Th1, Th2, and Th17 which are regulated reciprocally to maintain a balance in immune-mediated disease [19]. Regulatory T cells control adaptive immune responses by suppressing T cells, NK cells, NKT cells, B cells, and dendritic cells [20]. CD8^+^ T cells recognize antigen in association with MHC class I molecules and are predominantly cytotoxic. Cytotoxic T cells use various mechanisms to kill their targets, including direct cell-cell signaling via surface molecules and indirect signaling via cytokines.

### 2.2. Type 1 versus Type 2 DM

Type 1 diabetes is an organ-specific autoimmune disease characterized by a progressive cell-mediated destruction of pancreatic beta cells, leading to an absolute deficiency of insulin. Both activation of the T-cell-mediated immune system leading to insulitis and humoral B cell response producing immunoglobulins against beta cell autoantigens participate in the pathogenesis of type 1 DM [21, 22]. Developing a more aggressive T-cell phenotype and changing the Th1-to-Th2 balance towards a more proinflammatory milieu (Th1 dominant) may be associated with the progression towards overt diabetes. Furthermore, evidence demonstrating the association of the Th17 subset with pathogenesis of type 1 diabetes is rapidly accumulating [23–25]. By contrast, type 2 diabetes is a nonautoimmune form of diabetes characterized by insulin resistance and relative (rather than absolute) insulin deficiency. At present, however, little is known about the role of T cells in the process of insulin resistance, metabolic syndrome, or type 2 DM [26].

### 2.3. T Cells in Insulin Resistance, Metabolic Syndrome, and Type 2 DM

Adipose tissue inflammation is now recognized as a crucial process leading to the metabolic syndrome, diabetes and atherosclerotic cardiovascular disease [27–29]. However, how adipose inflammation is initiated and maintained is still unclear. Macrophage infiltration of adipose tissue has been described in both animal models and human diseases [27]. Accumulation of other immune cells, such as T cells, has been observed in obese adipose tissue recently [30–32]. T lymphocytes are known to interact with macrophages and regulate the inflammatory cascade [33]. Nishimura et al. performed studies to investigate the functional role of T lymphocytes in adipose inflammation [34]. In mice fed a high-fat diet, larger numbers of CD8^+^ effector T cells infiltrated obese epididymal adipose tissue, whereas the numbers of CD4^+^ helper and regulatory T cells were diminished. The phenomenon of infiltration by CD8^+^ T cells precede macrophage accumulation, and once CD8^+^ T cells were depleted using specific antibodies, macrophage infiltration, adipose tissue inflammation, and systemic insulin resistance were ameliorated [34]. Nishimura et al. also found that obese adipose tissue activates CD8^+^ T cells, which then promote the recruitment and activation of macrophages in this tissue [34]. These findings, indicating that systemic insulin resistance is ameliorated by CD8 depletion and aggravated by adoptive transfer of CD8^+^ cells, strongly suggest that CD8^+^ T cells play essential roles in the initiation and maintenance of adipose tissue inflammation and systemic insulin resistance [34]. 

Accumulation of CD8^+^ T cells in obese epididymal fat pads was not accompanied by the presence of greater numbers of CD8^+^ T cells in the systemic circulation, suggesting that CD8^+^ T cells are activated by endogenous stimuli localized in the adipose tissue [34]. Coincubation with CD8^+^ T cells plus lean adipose tissue may induce macrophage differentiation, suggesting that the interactions among CD8^+^ T cells, macrophages, and adipose tissue may activate and propagate a local adipose inflammatory cascade [34]. In contrast to CD8^+^ T cells, numerous CD4^+^ T cells and regulatory T cells were lower. The predominant T-cell effect on glucose homeostasis, revealed by conducting CD4^+^ T-cell reconstitution studies in lymphocyte-free mice, was the improvement of glucose tolerance, enhanced insulin sensitivity, and lessening of weight gain [35]. Regulatory T cells and subsets of CD4^+^ Th2 cells are known to secrete anti-inflammatory cytokines that can inhibit macrophage recruitment and activation [36]. Whether the reducing numbers of CD4^+^ and regulatory T cells augment the inflammatory response during the inflammatory cascades in obese adipose tissue requires further study to elucidate the detail mechanisms [34].

### 2.4. Effects of Hyperglycemia and Type 2 DM on T Cells

Elements of DM can directly or indirectly activate T cells. High-glucose concentrations may induce macrophage production of IL-12, which can stimulate CD4 cell production of IFN-*γ* [37]. By contrast, hyperglycemia may activate nuclear factor kB (NF-kB) through PKC and reactive oxygen species to rapidly stimulate the expression of cytokines [38, 39]. T lymphocytes from patients with diabetes which have an activated phenotype and TNF-*α*-expressing Th1 cells are prevalently detected [40–42]. The expression of IL-1, TNF-*α*, and macrophage migration inhibitory factor (MIF) is markedly upregulated in the injured kidney [43, 44]. Furthermore, longer disease duration results in increased advanced glycosylation end (AGE) products and AGE-modified proteins, which could bind to the receptor for AGE on macrophages and T cells, stimulating synthesis and release of proinflammatory cytokines in DM [45–47]. IFN-*γ* secretion by T cells can initiate and induce further inflammation and oxidative stress within renal tissues [47]. Advanced glycosylation end (AGE) induces synthesis of IFN-*γ* that further accelerates the inflammation by the activation of macrophages and vascular cells with renal tissues [42, 47].

## 3. Leukocyte Recruitment and Renal Injury in DN

Although DN has not been considered an inflammatory disease in the past and although metabolic or hemodynamic factors are the major causes contributing to DN, recent studies have suggested that DN is an inflammatory process, and immune cells might be involved in its development and progression [13, 48]. Diabetic nephropathy (DN) is an inflammatory disease with prominent leucocytes infiltratiing the kidneys. Most research has focused on the contribution of macrophages because they are the foremost infiltrating immune cells in diabetic kidneys [43, 49]. Inflammation induced by macrophages may constitute important mechanisms in the progression of DN [43, 49, 50]. The importance of macrophages in diabetic renal injury has been clearly demonstrated; however, little is known about the role of lymphocytes. The levels of circulating activated lymphocytes were higher in type 1 diabetic patients with proteinuria than those in nonproteinuria patients [40, 51]. This suggests that activated T lymphocytes may also be associated with the development of type 1 DN. Whether T cells are associated with the development of type 2 DN is still unknown.

### 3.1. Lymphocyte Recruitment in Diabetic Kidney

In patients with type 1 diabetes, T-cell influx and accumulation in the juxtaglomerular apparatus are the factors that exacerbate diabetes and correlate with glomerular filtration surface and albumin excretion rate [51]. Previous investigations have also shown folds increasing in glomerular and interstitial CD4^+^ and CD8^+^ T cells, as well as in interstitial FOXP3^+^ regulatory T cells in diabetic compared with non-diabetic wild-type mice [40, 51]. The development of early diabetic renal injury is associated with significant lymphocyte infiltration. There is no doubt that immune cells participate in the renal injury under the conditions of DN, and their migration into the kidney is a crucial step in the progression of this disease. Although the detailed mechanisms of leukocyte migration into renal tissues in DN are not completely understood nor is the functional role of T cells within this compartment, it has been reported that adhesion molecules and the chemokines are involved in this recruitment [42, 44, 52].

Recruitment of leukocyte is a key event in the disease progression of DN. Previous studies have demonstrated that mice deficient in intercellular adhesion molecule-1 (ICAM-1) may cause defects in macrophages and leukocytes homing into renal tissues, resulting in substantial reduction of renal injury [53]. The CD4^+^ T cells homing into glomeruli of diabetic kidney were decreased in ICAM-1-deficient-db/db mice, as compared with those of db/db mice [53]. Because naive and effector T cells constitutively express LFA-1, and ICAM-1 expression is found on renal endothelial, epithelial, and mesangial cells, it is likely that this interaction will plays a significant role during T-cell migration into the kidney [54–56]. Chemotactic cytokines are also major factors that induce the recruitment of inflammatory cells into the kidney, subsequently amplifying the immune-mediated damage [57]. Once macrophage is infiltrated within the diabetic kidney, the macrophages and macrophage-derived products can induce further inflammation [43]. Monocyte chemoattractant protein-1 (MCP-1), an important chemokine regulating macrophage recruitment, is upregulated in patients with DN [58]. Moreover, constitutive RANTES expression directs subset-specific homing of CD4^+^ T cells in the kidney [59]. The role of RANTES in directing T lymphocyte homing into the diabetic kidney is not yet clear.

Compared to Type 2 diabetic patients without DN, significantly lower plasma concentrations of sCTLA-4 and higher concentrations of sCD28 were noted in Type 2 diabetic patients with DN [60]. Furthermore, plasma sCD28 and sCD80 were found to be positively correlated with the fasting urine albumin, creatinine ratio in DN patients. The disease severity of DN related with elevated soluble adhesion molecule vascular cell adhesion molecule-1 and P-selectin were also found [60]. Costimulatory molecules, together with leukocyte adhesion molecules, are crucial for T lymphocyte and leukocyte-mediated inflammatory responses. The aberrant expression of soluble costimulatory molecules and adhesion molecules may be related to the activation of T cells and leukocytes in the progression of inflammation in type 2 DN [60].

### 3.2. Leukocytes and Diabetic Nephropathy: Cause or Consequence?

Increased infiltration of monocytes/macrophages and activated T lymphocytes, as well as augmented expression of inflammatory cytokines in the kidneys, have been found in patients with DN [9–11]. Longer disease duration of DM results in an increase of advanced glycosylation end (AGE) products and AGE-modified proteins that may bind to leukocytes, stimulating the synthesis and release of proinflammatory cytokines in DM [45, 46]. By contrast, an activated renin-angiotensin-aldosterone system (RAAS) and endothelial dysfunction, well noted in patients with DM, have also been proven to be a crucial determinants of leukocyte activation and cytokine expression in generating proinflammatory and proliferative effects [61–63]. Thus, it is highly possible that metabolic or hemodynamic factors in DN may trigger the immune-mediated inflammatory responses and cytokine production. Furthermore, prominent leukocyte infiltration in models of remnant kidneys or unilateral ureter obstruction, models considered to be nonimmunologically mediated, was also illustrated. Hence, the accumulation of leukocyte in the kidneys of DN can be the results of lesions from high glucose or glomerular hyperperfusion (secondary to hyperglycemia or hypertension), rather than the cause of DN. Therefore, leukocytes may be either the cause or the consequence of DN.

### 3.3. Interaction and Mechanisms of T-Cell-Mediated Renal Damage

The scale of CD4^+^ and CD8^+^ T cells accumulated in the diabetic kidneys was much smaller than that of macrophages in rodent models of both Type 1 and Type 2 DN [43, 64], suggesting that T cells may interact with macrophages to regulate inflammation and renal injury. Activated T cells can cause injury directly through cytotoxic effects and indirectly by recruiting and activating macrophages. Proinflammatory cytokines secreted by T (CD4^+^, CD8^+^) cells could activate neighboring macrophages directly or by stimulating mesangial cell production of colony stimulating factor-1 and MCP-1 indirectly [65]. Once macrophages have activated, they can release nitric oxide, reactive oxygen species, IL-1, TNF-*α*, complement factors, and metalloproteinases, all of which promote renal injury [49, 50]. T cells express the receptor for AGEs and can respond to AGEs [47]. The activation of CD4^+^ and CD8^+^ T cells by AGE can initiate IFN-*γ* secretion by T cells [47], which could induce further inflammation and oxidative stress within the diabetic kidney. In addition, CD8^+^ cells may perform a cytolytic function in the diabetic kidney. All of these cytokines and molecules promote inflammation and induce further expression of macrophage colony-stimulating factor and ICAM-1 in renal cells, further contributing to renal injury [48, 66]. 

Nevertheless, infiltrating macrophages and T lymphocytes are the most probable sources of many cytokines mediating the renal injury in DN and its progression. The intrinsic renal cells (endothelial, mesangial, glomerular, and tubular epithelial cells) are able to synthesize many pro-inflammatory cytokines [67, 68]. At high glucose levels, podocytes are considered the major sources of IL-1*α* and IL-1*β*, and they may also produce MCP-1 [69, 70]. Increased secretion of TGF-*β* by peripheral blood mononuclear cells has been reported in patients with DN and seems to be responsible for fibrogenic and proliferative effects on fibroblasts [71–73]. Furthermore, the TGF-*β* is also a crucial pleiotropic cytokine associated with the development of Tregs and Th17 cells [19]. Collectively, induction of proinflammatory and profibrogenic molecules influences renal damage in diabetes. 

The aberrant production of inflammatory cytokines and chemokines, as well as differential activation of MAPK in different leucocytes (T helper (Th) cells and monocytes), are the underlying immunopathological mechanisms of type 2 DM patients with DN [74]. An increasing body of evidence indicates that immigrated blood leukocytes might considerably alter the phenotype of endothelial cells and increase inflammation of the vascular bed [75]. Endothelial dysfunction is associated with most forms of cardiovascular disease, such as coronary artery diseases, chronic renal failure, and diabetes [76]. Interaction of renal tissue macrophages and T cells produces various reactive oxygen species, proinflammatory cytokines, metalloproteinases, and growth factors, which modulate the local response and increase inflammation within the diabetic kidney [5, 8, 77, 78].

## 4. Roles of T Cells in Type 2 Diabetic Nephropathy

### 4.1. Th1 Cells

The circulating lymphocytes trafficking through tissues may interact with tissue AGEs. Exposure of activated T lymphocytes to AGE may enhance the expression of interferon gamma (IFN-*γ*) indicating that the T cell AGE-receptor system might be linked to lymphokine production involving in renal damage. Under conditions of excessive AGE-protein and AGE lipid accumulation (e.g., aging and diabetes), enhanced production of AGE-induced IFN-*γ* may accelerate immune responses that contribute to tissue injury.

It is well recognized that T helper-1 (Th1) response precedes and accompanies type 1 diabetes [21]; hence, it is possible that Th1 cells are prevalent in type 1 diabetic kidney. Elevated levels of ICAM-1 and P-selectin within the diabetic kidney, combined with increased levels of IFN-*γ* and MIF, were associated with the homing of effector Th1 cells in glomeruli [79]. However, little is known about the mechanisms of Th1 cell migration in the type 2 DN model during the development and progression of kidney diseases. Higher serum IFN-*γ* levels, a Th1 cytokine, and positive correlations between plasma IFN-*γ*, proteinuria, and estimate glomerular filtration rate (eGFR) were found in type 2 diabetic patients with overt nephropathy [80]. Plasma IL-2R levels found in type 2 DM patients with overt DN were higher than those without overt nephropathy. Furthermore, a significantly positive correlation was determined between plasma IL-2R and proteinuria [80]. These results indicate that Th1 cellular immunity in conjunction with Th1 and proinflammatory cytokines may mediate tissue injury in patients with DN [80].

### 4.2. Th2 Cells

Th2 cells, producing IL-4 cytokines, can contribute to humoral immunity, suppress Th1 cell activation, and function as an inhibitory cytokine of autoimmunity and inflammations [17]. No significant change of serum IL-4 level in type 2 DN patients, as compared to those without nephropathy [80]. Specific polymorphisms within the IL4R locus and by specific genotypes at the IL4R, IL4, and IL13 loci were strongly associated with susceptibility to type 1 DM [81]. Furthermore, an association of interleukin (IL)-4 intron-3 polymorphism with susceptibility to end-stage renal disease was discovered [82]. However, the role of IL-4 gene polymorphisms in type 2 diabetic nephropathy still requires further evaluation.

IL-10, another important Th2 cytokine, exerts predominantly anti-inflammatory and immunosuppressive effects [83]. Low production capacity of IL-10 associated with the metabolic syndrome and type 2 DM [84]. Some studies have revealed elevated IL-10 levels in the sera of diabetic patients with nephropathy, and a positive correlation between IL-10 levels and albuminuria has been suggested to participate in the DN pathogenesis [85–87]. IL-10 promoter variants and haplotypes (GTA and GTC) have predictive value in determining the susceptibility to nephropathy in Tunisian T2DM patients [88, 89].

### 4.3. Th17 Cells

Th17 is a third distinct subset of T helper cells and have been found to play vital roles in the pathogenesis of several autoimmune diseases such as multiple sclerosis and rheumatoid arthritis [19]. Increasing evidence demonstrates that the Th17 cell in type 1 DM in murine model and human type 1 DM [23–25]. Recently, T cell in type 2 DM patients has been revealed to be skewed toward a proinflammatory phenotype, requiring monocytes for maintenance and promoting chronic inflammation through elevated IFN-*γ* and IL-17 production [90]. However, IL-17A cannot be concluded to be associated with nephropathic complications of type 2 DM, due to an increased serum level of IL-17A found in patients with nonnephropathy [91].

### 4.4. Treg Cells

The expression rate of CD4^+^CD25^+^Foxp3^+^ Treg cells between the control group and type 2 diabetic patients yielded no significant difference [92]. In type 2 diabetic patients with microalbuminuria and macroalbuminuria, the expression of CD4^+^CD25^+^Foxp3^+^ Treg cells was significantly lowered, as compared with that of the control group, and patients with macroalbuminuria showed significantly lower expression of CD4^+^CD25^+^Foxp3^+^ Treg cells than did the microalbuminuric patients. Significant inverse correlations were noted between the disease course and the expression of CD4^+^CD25^+^Foxp3^+^ Treg cells between the urinary albumin excretion rate (UAER) and the expression of CD4^+^CD25^+^Foxp3^+^ Treg cells. Whether the CD4^+^CD25^+^Foxp3^+^ regulatory T cells (Treg) play role in type 2 DN still requires further investigation [92].

### 4.5. Cytotoxic T Cells

Adipose tissues can activate CD8^+^T cells, which then promote the recruitment and activation of macrophages resulting in the metabolic syndrome, which can be significantly attenuated by a specific CD8 antibody [34]. In the diabetic kidneys of NOD mice, accumulation of CD8^+^ cells is associated with increased expression of genes encoding perforin and granzyme B, as well as with colocalization in immunostaining for perforin [93]. This suggests that CD8^+^ cells may perform a cytolytic function in the diabetic kidney. In streptozocin-induced diabetic nephropathy, previous studies have shown that the difference in expression of CD4^+^ T cells in control and diabetic kidneys is more significant at 1 month than at 8 months, whereas expression of CD8^+^ T cells is more significant at 8 months. It is speculated that DN is probably initiated and driven by a Th1 process. The function of CD8^+^ T cells, however, becomes more significant at later stages of the disease when tissue loss is evident [94].

## 5. Type 2 DN and Therapeutic Strategies Involving T Cells

Due to the pathogenic complexity of DN, protecting diabetic patients from the development and progression of renal injury remains a challenge for physicians. The accumulation of inflammatory cells in renal biopsies of diabetic patients is associated with tissue damage and a progressive decline in renal function [95]. In animal models, the use of immunosuppressants, neutralizing antibodies, and genetic deficiencies has shown that reducing leucocyte accumulation and activation in diabetic kidneys suppresses the development of renal injury [96]. Many previous studies have used anti-inflammatory strategies that also suppress accumulation of lymphocytes in diabetic kidneys, indicating that lymphocytes may also contribute to disease progression. Inflammation plays an important role in the pathogenesis of proteinuria in DN. Inhibiting renal macrophage recruitment by immunosuppressant therapy, or modulating of MCP1 or ICAM-1 expression in diabetic mice, demonstrate anti-proteinuric and renoprotective effects [13, 69, 97–99].

The mammalian target of rapamycin (mTOR) is a serine/threonine kinase that plays a pivotal role in mediating cell size and mass, proliferation, and survival [100]. High activation of mTOR within the kidney has been reported to occur in DN [101, 102]. Systemic administration of rapamycin, a specific and potent inhibitor of mTOR, markedly ameliorated pathological changes and renal dysfunctions in *db/db* mice [102]. Rapamycin markedly inhibited the influx of inflammatory cells, predominantly lymphocytes, and macrophages, associated with DN [100–104]. This effect is likely attributable to rapamycin-induced inhibition of the proliferation and clonal expansion of B and T lymphocytes [103–105]. Within the kidney, rapamycin also ameliorates the release of proinflammatory cytokines and chemokines, such as monocyte chemoattractant protein-1, RANTES, IL-8, and fractalkine, exacerbating the inflammatory process in DN [103–105]. These results indicate that mTOR activation plays a pivotal role in the development of DN and that rapamycin could be as a strong therapeutic potential agent for DN.

Depletion of CD8^+^ T cells using specific antibodies could ameliorate macrophage infiltration, adipose tissue inflammation, and systemic insulin resistance. Whether depleting CD8^+^ T cells could be applied in treatment of DN is still undetermined. Anti-inflammatory or immunosuppressive treatment has been applied to treat DN in animal models [106–109]; however, therapeutic approaches targeting T cells are still limited.

## 6. Conclusion

Beyond traditional metabolic and hemodynamic risk factors, type 2 DN is now increasingly considered as an inflammatory disease. The inflammatory process is not only due to an innate immune response dominated by macrophage-mediated effects, but also by the adaptive immune response mediated by leukocytes. T cells participate in the development of type 2 DN from processes of insulin resistance, metabolic syndrome, and type 2 DM into type 2 DN. The recruitment of leukocyte is a key event in the disease progression of DN. Diverse immune cells and cytokines exert important roles in the pathogenic complexity of development and progression during the DN process. Interaction of renal tissue macrophages and T cells produces various reactive oxygen species, proinflammatory cytokines, metalloproteinases, and growth factors, which modulate the local response and increase inflammation within the diabetic kidney. A better understanding of the role of T cells in the context of DN will create several new opportunities for therapeutic intervention that may benefit patients with DN.
